# Using argument notation to engineer biological simulations with increased confidence

**DOI:** 10.1098/rsif.2014.1059

**Published:** 2015-03-06

**Authors:** Kieran Alden, Paul S. Andrews, Fiona A. C. Polack, Henrique Veiga-Fernandes, Mark C. Coles, Jon Timmis

**Affiliations:** 1 York Computational Immunology Laboratory, University of York, York, UK; 2 Centre for Immunology and Infection, University of York, York, UK; 3 Department of Computer Science, University of York, York, UK; 4 York Centre for Complex Systems Analysis, University of York, York, UK; 5 Department of Electronics, University of York, York, UK; 6 Faculdade de Medicina de Lisboa, Instituto de Medicina Molecular, Lisboa, Portugal; 7 SimOmics Ltd, The Catalyst, Baird Lane, Heslington, York, UK

**Keywords:** computational modelling, argumentation, simulation, Artoo, immune system modelling

## Abstract

The application of computational and mathematical modelling to explore the mechanics of biological systems is becoming prevalent. To significantly impact biological research, notably in developing novel therapeutics, it is critical that the model adequately represents the captured system. Confidence in adopting *in silico* approaches can be improved by applying a structured argumentation approach, alongside model development and results analysis. We propose an approach based on argumentation from safety-critical systems engineering, where a system is subjected to a stringent analysis of compliance against identified criteria. We show its use in examining the biological information upon which a model is based, identifying model strengths, highlighting areas requiring additional biological experimentation and providing documentation to support model publication. We demonstrate our use of structured argumentation in the development of a model of lymphoid tissue formation, specifically Peyer's Patches. The argumentation structure is captured using Artoo (www.york.ac.uk/ycil/software/artoo), our Web-based tool for constructing fitness-for-purpose arguments, using a notation based on the safety-critical goal structuring notation. We show how argumentation helps in making the design and structured analysis of a model transparent, capturing the reasoning behind the inclusion or exclusion of each biological feature and recording assumptions, as well as pointing to evidence supporting model-derived conclusions.

## 1. Introduction

The application of computational and mathematical models is a generally accepted technique in physical sciences and engineering, yet was met with scepticism by experimental biologists [1]. With advances in computational power, the development of new modelling techniques and methodologies, and the promotion of interdisciplinary research, traditional biological studies complemented by the use of *in silico* predictive tools is now becoming more prevalent. Computational models have the capacity to provide an interpretation of biological data and act as a scientific tool through which new hypotheses can be established and explored [2,3]. Furthermore, computational tools allow virtual experimentation not bound by the ethical and financial constraints associated with laboratory studies.

Computational techniques can appear to be opaque tools that provide a researcher with a result or prediction but little understanding of how the result has been reached [4]. The application of scientific software continues to be discussed in journals [5,6], with concerns that researchers place too much trust in published software, and suggestions that code, as well as methods and results, should be subject to peer review [7]. The concern about adequacy of software engineering is appropriate, but the same focus needs to be applied to the underlying biological information from which a computational model is been constructed. In the course of implementation, decisions are made concerning the interpretation of available biological data, how that biological data will be translated into a form that can be effectively expressed on a computer; and which assumptions or abstractions are to be employed to mask gaps in the biological understanding. An appreciation of the underlying biological information and the ways in which it is used in the computational model is critical to the sensible interpretation of computational results in the context of the biological domain. However, it is rare to see a published model of a biological system accompanied by any in-depth description or justification of the decisions made in development. While much focus has been given to the release of software tools that aid researchers in developing and analysing computational models [8–13], the same attention has not been given to providing researchers with a means of showing that their developed tool can adequately support the investigation of a specific biological research question: that the tool is fit for purpose.

We have previously developed a computational model, or simulation, of pre-natal lymphoid tissue formation to help direct and understand results of laboratory experimentation [14,15]. Through the use of a cell culture system, the behaviour of cells was tracked for a period of 1 h, after which key cell behaviour responses were calculated. These responses revealed that there is a statistically significant change in cell behaviour when in the vicinity of developing lymphoid tissue [14]: the reasons for which are currently unclear. Our computational model adopts an agent-based approach, allowing exploration of how system dynamics, in this case changes in cell behaviour responses, might emerge from interactions between cells and their environment [15,16]. For the full detail of our implementation, we direct the reader to our previous descriptions of the biological information captured in the model, and the manner by which this has been translated into a specification that can be encoded as a computer program and simulation platform [14,15]. As a brief overview for the purposes of this paper, three cell populations are known to be involved in lymphoid tissue development, counts of which have been calculated from flow cytometry experiments. Other attributes, such as cell speed, have been determined from either laboratory experiments or from the literature. Our model captures each of these cells: each of which possesses individual attributes and state. Transitions between these states are described in a set of rules, described in detail in unified modelling language (UML) state diagrams [15]. With each simulation time-step, cell behaviour is simulated dependent on the current state of the cell and the cell's location. The environment, in this case the developing gastrointestinal tract, is modelled as a continuous space, with dimensions set that are representative of measurements taken from stereomicroscopy images. Simulated adhesion and chemokine diffusion pathways influence the behaviour of these cells, causing the emergence of aggregations of cells within the simulated environment: aggregations that become lymphoid organs. Our previously published studies demonstrate that we could use simulation to reproduce emergent cell behaviour that is statistically similar to that observed in *ex vivo* culture, thus providing us with a strong baseline behaviour from which we can use the tool to explore the mechanisms underlying tissue development [14,15]. Through careful statistical analysis of simulation behaviour, including sensitivity analyses [13], we were able to identify key pathways in the simulated model and suggest how the existence of such pathways in the biological system could be investigated in the laboratory. However, as for any model, the simulation and its results are heavily influenced by the implementation decisions taken when our simulator was developed.

In general, simulations of complex systems, including biological systems, are difficult to describe: it is hard to explain and justify complex interactions, either in real biological systems or in simulations. Toxicology and human risk assessment studies, for example, use adverse outcome pathway (AOP) tools to demonstrate existing understanding of how molecular, cellular, organ and organism interactions link a molecular initiating event with a particular adverse outcome, such as skin inflammation [17]. This information, derived from the literature or experimental studies, is analysed and presented as a flow diagram. The strength of the evidence supporting each event, which may be established as well as hypothetical or predictive, is evaluated and accompanies the diagram. Yet, AOPs have been criticized for providing a representation of the toxicological process that is simplistic [17], splitting the representation of the process and the evidence. For the description of computational models, both unified modelling language, adopted in the development of computer software, and systems biology mark-up language (SBML) may be applied [18–20]; the latter possessing the benefit of allowing model execution by a number of SBML-supported software tools. Both however are limited by restrictions in the extent of the system they can capture: UML lacks the formalism to capture some biological features (such as cyclic-feedback) [21], and SBML cannot currently describe complex agent-based models. In addition, both are purely descriptive: neither provides complete, evidence-supported detail stating how that model has been composed. In ecology, the ODD (overview, design concepts, details) protocol is starting to address this, through application of a standard protocol completed while implementing a computational model, with the aim of ensuring reproducibility of results [22]. The ODD protocol addresses the purpose behind the creation of the model, details the inclusion of each biological component of interest (e.g. cell type) and defines submodels that describe how observed biological behaviour and attributes are implemented. Any specific assumptions underlying the implemented behaviour are also recorded. The ODD authors note that completion of the protocol provides researchers with all the information they require to run the simulation and reproduce the published results: the level of information required for a typical methods section of a publication [22]. Yet, ODD also does not provide a motivation and justification for the detailed model and implementation. Scientific repeatability is addressed, not fitness for purpose. The ODD authors also state that, within the protocol, there should be no recording of information concerning experimental scenarios, simulation experiments and results from statistical techniques such as sensitivity analyses: these should be recorded and published separately [22]. However, we contend that having such information within the simulation design is a key part of an argument of fitness for purpose that convinces researchers the simulation is appropriate for the studies in which it will be applied.

Here, we present the use of a structured argumentation approach that assists the researcher in recording justifications and rationale for both the biological detail and engineering process that underlie the development of a computational model, to assist others in interpreting the predictions that model generates. We suggest the adoption of this approach within an existing process of computational model development, for example that suggested in [3,15], yet acknowledge the potential for a researcher to use constructed arguments as a guide for alternative processes of model development. The argumentation approach is widely applicable and is independent of the choice of techniques used for model description: it can be applied to models described in notations such as UML or SBML and to models accompanied by ODD or AOP descriptions. Our approach takes inspiration from the field of safety-critical systems: like biological systems, safety-critical systems comprise complex interactions, within a system, between systems and with the wider environment. In critical systems engineering, a system cannot be declared safe, but can be demonstrated to be ‘as safe as reasonably practicable’ [23]. Prior to the adoption of safety-critical systems (e.g. aircraft, certified by military or civil aviation authorities), it must be shown that the system and its development meet stringent compliance requirements: hazards must be shown to have been identified systematically and thoroughly, and mitigated to an appropriate level. Acceptable safety can be established and presented using arguments over evidence. We have adapted this technique to give a structured argumentation approach that can be used to demonstrate acceptable fitness for purpose. In common with acceptable safety, our argumentation approach aims to capture and expose reasoning, via an argumentation structure, to critical scrutiny, in order to establish trust in simulations [24,25]. While it may seem unnecessary to use techniques from aircraft safety in biological simulation, we note that errors or misinterpretation of simulation results may have significant, possibly even safety-critical repercussions if, for instance, a simulation is used as a key decision-making tool in clinical trials [26,27]. Opening all simulation design and implementation decisions to critical scrutiny requires that elements captured in the model be traceable to the biological domain, and their inclusion justified with respect to abstraction levels and the purpose of the simulation.

Drawing on safety-case argumentation, we create a diagrammatic summary of the structured argument of fitness for purpose, using a visual notation closely based on the standard safety-critical argumentation notation, goal structuring notation (GSN) [28,29]. The argument is presented as a tree of connected argument components, starting from a top-level claim (a GSN goal). The researcher identifies a set of fitness-for-purpose requirements (referred to as goals or claims, that the argument seeks to substantiate), and a set of strategies that can be used to assess whether the requirement has been met. The strategies typically break goals down into subgoals, and eventually link to evidence supporting the claim, alongside the source of the evidence, where appropriate. In the simulation context, the GSN argument structure summarizes the biological information upon which the model has been constructed, opening this to critique. GSN also allows goals to be linked to assumptions and justifications. If a requirement cannot be fully supported by available evidence, for example where there are gaps in the biological understanding, then the assumptions and abstractions made in place of this evidence are documented, opening all implementation decisions to scrutiny by other researchers in the field and identifying areas of biological study that have been overlooked or require further laboratory work. In addition, and in contrast to the ODD protocol described above, argumentation approaches can be applied not only to simulation development, but also to design and sensitivity analyses and simulation experiments.

One notable side effect of focusing on the justifications for a simulation model is to engage researchers from differing disciplines in the process of capturing the model to be implemented. Following sound software engineering principles, we can express the model in structures and language that is not discipline dependent, and then provide an argument that the implementation conforms, in a traceable, repeatable manner, to this model as part of the overall fitness-for-purpose argument [30]. Constructing the argument using a visual notation results in a document that can be easily interpreted by researchers across disciplines, and which can be published alongside the description of and results from the simulation. The overarching objective of this approach is to increase confidence in the use of simulation-derived predictions, potentially increasing the impact of a simulation study. While it is possible that providing a detailed rationale behind simulation development might risk limiting a researcher's willingness to challenge the results of work supported by argumentation, we feel this technique has the potential to address an important void in biological simulation development: the provision of a method by which a researcher can fully appreciate the thought processes behind model composition. In biological contexts, the rationale is never a fully explored, uncontentious argument, because the context is not that well understood. Instead, the argument exposes the rationale and understanding to critique, initiating a healthy debate about the quality of the experimentation and the results.

To support both our adoption of the argumentation approach and to encourage wider use of argumentation in conjunction with simulation-based research, we have developed a freely available Web-based argumentation tool, Artoo (www.york.ac.uk/ycil/software/artoo). Here, we demonstrate argumentation, and the use of Artoo, by presenting the argumentation that supports our simulation of lymphoid organ development [13–15]. We present our implementation decisions and expose to scientific scrutiny the case that our simulation is fit for the defined purpose of our published study. We demonstrate the power of the approach in providing an accessible description of the detail and rationale of a simulation of a biological system, ensuring anyone using our model can see clearly how the biological information has been used and can assess the impact our assumptions and abstractions have on simulator response. The Artoo tool, the description of the GSN-like notation and the case study together make it possible for researchers to adopt the argumentation technique, with the objective of increasing confidence in the application of computational modelling and predictive tools.

## 2. Material and methods

### 2.1. The biological case study: lymphoid tissue development simulator

Through the adoption of an agent-based modelling approach, we have developed a computational model, or simulation, of lymphoid tissue development in pre-natal mice [14,15]. Populations of haematopoietic cells migrate into the developing gut from embryonic day 14.5, forming aggregations of cells around adhesion factor expressing stromal cells 72 h later [31–33]. These aggregations mature to form lymphoid organs capable of triggering adaptive immune responses to pathogens. Cell observations captured in *ex vivo* culture at hour 12 of development (velocity and displacement) reveal that haematopoietic cells behave in a statistically significantly different manner when in the vicinity of a developing aggregation [14]. Although a basic model of tissue formation has been developed through laboratory experimentation [31,34–36], the reasons for this emergent behaviour are not fully understood. Adopting an agent-based modelling approach has allowed us to simulate the behaviour of each individual cell within a simulated gastrointestinal tract environment, with each cell possessing attributes (cell speed, size, etc.) and behavioural characteristics observed in the laboratory or in the literature. Data analyses suggest that there is no statistical difference between the emergent behaviour of cells in the *ex vivo* culture system and our simulated haematopoietic cells [14]. This result, combined with a rigorous statistical analysis of simulation behaviour [13] has provided us with a tool that we have used to develop and test hypotheses that can inform future laboratory investigations.

### 2.2. The argumentation tool: Artoo

**Figure 1.**
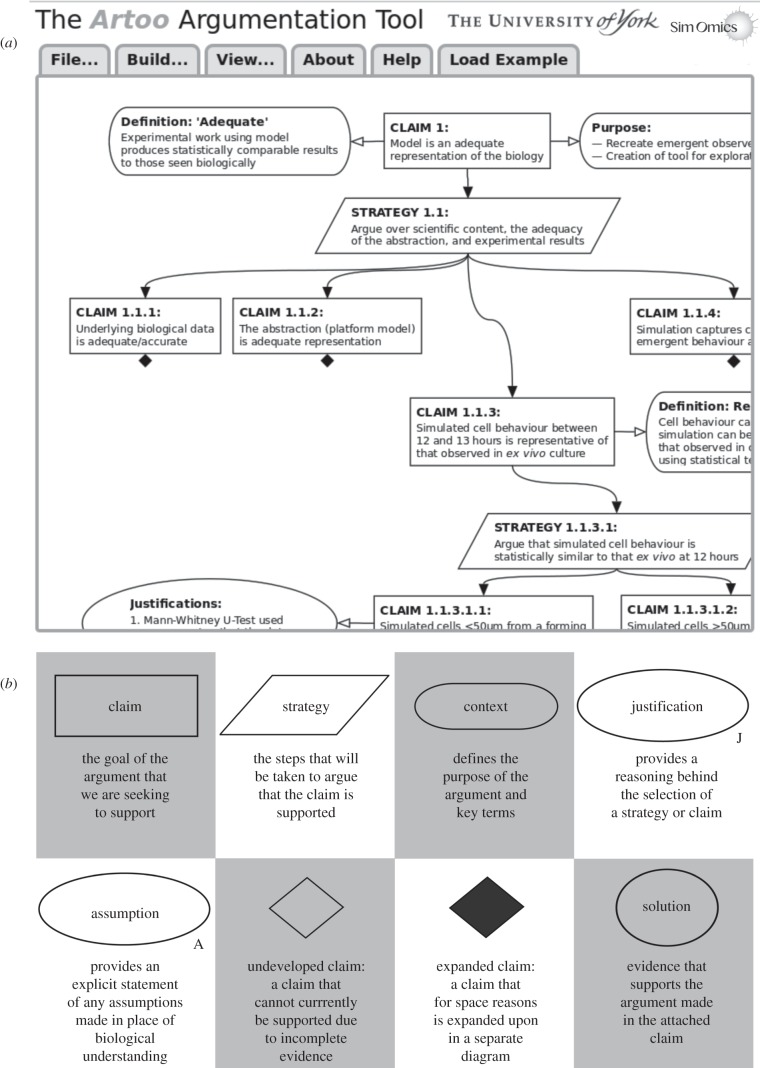
(*a*) A screenshot of the Artoo argumentation tool. This runs in a Web browser window in an up-to-date version of either Chrome or Firefox browser (other browsers do not fully support the required technologies). The File menu provides three options: open a previously developed Artoo argument structure; save an argument structure, export the current argument structure as a PNG image. The Build menu provides options to create, edit and delete nodes (representing argument components); and to create or delete connections among nodes. The View menu operations enable zooming in, zooming out and centring of the argument structure. Nodes in the argument structure can be individually moved using the computer mouse by left clicking and dragging the node. Left mouse clicking and dragging on areas outside of the nodes will drag the entire structure. Right mouse clicking on an argument node (shown in the screenshot on the ‘Assumptions' node) allows access to a node-specific menu to edit or delete the node, add or delete a connection to that node, or collapse and hide the nodes below this node in the tree. A collapsed section of an argument is denoted by a black diamond symbol. (*b*) Definitions of each node type available in goal structuring notation.

Artoo was developed to provide an argument-driven platform for presenting a stringent analysis that the design and implementation of a computational model of a biological system is fit for purpose (figure 1). This permits the construction of an hierarchical argumentation structure using a GSN-like, graphical notation. The tool released is under a GNU GPL3 licence, is freely accessible via www.york.ac.uk/ycil/software/artoo and runs in an up-to-date version of the Chrome and Firefox browsers. To encourage adoption of the approach, full instructions are available alongside the tool and the argumentation structures in this paper can be loaded into the argumentation design window for further exploration. Argumentation structures developed in Artoo can be extracted as high-quality PNG images that can be published alongside a description of a developed simulation. Evidence that may be located elsewhere, such as in publications or in statistical analyses, can be embedded within the structure through use of hyperlinks, making all the argument information available to a researcher analysing the implementation.

### 2.3. Constructing an argument

An argument is developed as a hierarchical decomposition of claims relating to the purpose of the simulation. The goal of the argument is to support a claim, ultimately with evidence if possible, while keeping a record of the context of that claim.

The first step in constructing any argument is to identify the top-level claim that we are seeking to establish and its context. The context of a top-level claim in a fitness argument needs to clarify the purpose of the simulation and define key terms. The context is connected to the claim with lines with white arrowheads. Components supporting the top-level claim, explained next, are connected with lines with solid arrowheads.

Connections between a top-level claim and supporting subclaims are made via a strategy node. This strategy should detail the steps that will be taken to argue that the top-level claim is supported. These strategies may in turn lead to the definition of further claims, subclaims, that are then argued in turn. Examining a subclaim increases confidence that its parent claim holds, and all subclaims are considered together when making an overall judgement on whether the top-level claim is met. Some subclaims can be substantiated by pointing to evidence, such as statistical results or published research. Whereas in safety-critical safety cases, where all claims must ultimately be substantiated by evidence, fitness arguments allow us to mark some *undeveloped claims*. This allows us to indicate uncertainties: areas where the biological knowledge is missing or incomplete, or where further biological experimentation can be usefully undertaken. Undeveloped claims should not be seen as a weakness in the simulation, but as a clear statement that the researcher has recognized a gap in understanding, and addressed these in a particular manner (through use of assumptions or an abstraction). Claims that cannot be substantiated are accompanied by a white diamond.

When each identified claim and subclaim has been documented, the argument structure summarizes how the researcher believes the top-level claim can be substantiated. Reviewers and collaborators can peruse the structure to gain an appreciation of the link between the biological system and the simulation, and to identify any areas where the simulation authors can be challenged to improve their argument [37].

## 3. Results

### 3.1. Arguing fitness of the lymphoid development simulator using Artoo

**Figure 2.**
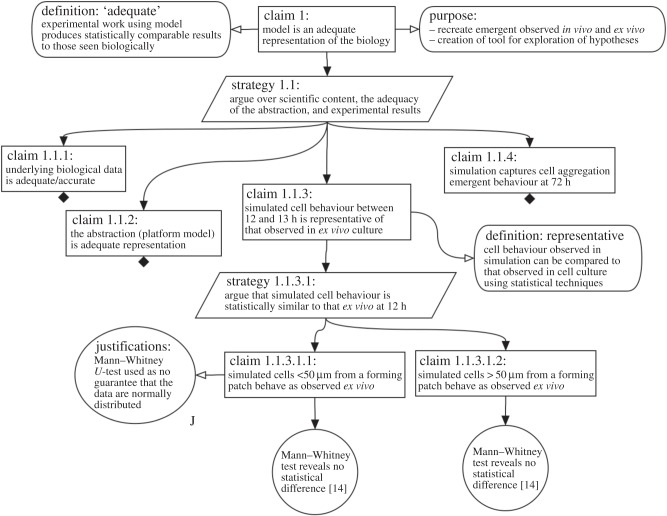
Top-level of argumentation structure used during the development of a computational tool that captures Peyer's Patch development, as output by our Artoo. The tool enables the developer to capture their claims and evidence using GSN. A claim is made that the simulation is an adequate representation of the biology, and arguments stated that support this claim. In turn, this claim is split into four subclaims. A black diamond notes that the claim has been developed yet is shown in the following figures due to limitations on space. Claim 1.1.3 has been developed in this figure, noting the evidence that simulated cell behaviour at the 12 h time-point is statistically similar to that observed *ex vivo*. Where a goal is stated, the tool enables the developer to link the goal to the evidence that supports the claim. Here, the figure is demonstrating that this evidence is within selected publications.

We present our argument that our lymphoid tissue development simulation [14,15] is an adequate representation of the biological system, and thus fit for the purpose of studying haematopoietic cell aggregation. Figure 2 shows the top-level claim, that ‘our model is an adequate representation of the biology’, and its immediate subclaims. To begin the process of arguing that this is the case, we explicitly state what we mean by an adequate representation and state the purpose behind the implementation of the model: a vital consideration when determining what simulation results mean in the context of the biological system. The rationale for substantiating the top-level claim is stated in the attached strategies. Here, we argue over ‘the scientific context, the adequacy of our abstraction and adequacy of experimental results'. In this case, the strategy leads to four subclaims that explore: the underlying biological data upon which our simulation is based (claim 1.1.1); the evidence supporting any abstractions that have been made (claim 1.1.2) and the ability of the simulator to reproduce cell behaviour observed in the laboratory and detailed in the literature (claim 1.1.3 and 1.1.4). The arguments supporting each of these claims can be developed separately.

### 3.2. Arguing adequate representation of the biology

Figure 2 shows the expansion of claim 1.1.3, that our simulated cell behaviour is representative of that observed in the laboratory. This is a key claim in our argument: we are presenting evidence to support our belief that the cell behaviour that emerges in the simulation is representative of the biological system. Similarly to the top-level claim, the subclaim is accompanied by a strategy that was used to support that claim (strategy 1.1.3.1). As noted in our description of our simulator in the Methods, through use of an *ex vivo* culture system we have obtained cell behaviour observations to which simulated cell responses can be contrasted [14]. We make further subclaims in figure 2 that the behaviour captured in this system is statistically similar to that observed in simulation, for both cells close to (less than 50 µm) and far from (more than 50 µm) a forming aggregation of haematopoietic cells. For full transparency, we state the statistical test that we have used in this comparison, and a justification of why we believe that this is the correct statistical test for drawing this conclusion. Finally, we show the evidence that supports the structure of claims (in circles): Mann–Whitney *U*-test statistics that reveal no statistical difference between the behaviour of cells in the simulation and that in the laboratory, with a note stating where the biological data we have used to draw this conclusion can be located. In Artoo, hyperlinks can be used to link to such supporting evidence.

### 3.3. Biological data: availability, adequacy and accuracy

**Figure 3.**
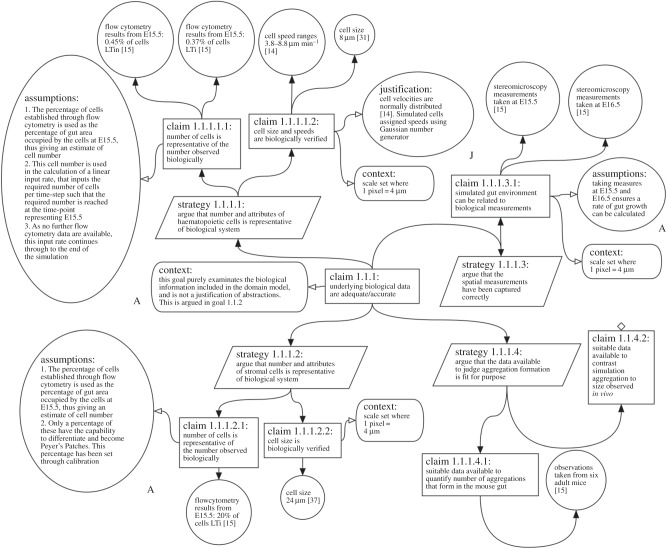
Expanded argumentation structure for claim 1.1.1 in figure 2. The argument was created during the development of a lymphoid tissue development simulator that captures Peyer's Patch development. The claim argues that the biological data against which the simulation is judged are adequate. Where a claim is stated, the tool allows the developer to link the claim to evidence that supports the claim. In the majority of cases, evidence is provided to substantiate the claim. However, where data are unavailable, the claim cannot be substantiated, shown by a blank diamond.

Whereas claim 1.1.3 above details how the simulation result compares with specified biological data, claim 1.1.1 (figure 3) examines the adequacy and accuracy of the biological data upon which our simulation has been constructed. The argument summarizes the specific biological data that has been used in the implementation, the source of these data, and where applicable, assumptions relevant to that data.

The claim in figure 3 is supported from evidence generated from four argumentation strategies, each concerning a subset of simulation parameters derived from biological data: properties of haematopoietic (LTin/LTi) cells; properties of stromal (LTo) cells; representation of the intestine environment and haematopoietic cell aggregation characteristics. The objective of the argument under this claim is to document the link between the simulation implementation and the real-world domain. Thus, available flow cytometry data, stereomicroscopy measurements and data mined from the literature are all included to support subclaims that the simulation has been built on appropriate data. The argument ensures it is clear how we have used these biological data to derive simulation parameters. For example, we explain how we convert flow cytometry data into estimates of cell numbers through the time course of the simulation. This record of what data were used, and how, is an important information for a researcher to have when conducting a full statistical analysis of the effect that a parameter has on a simulation response.

As previously noted, there are many interesting biological questions for which no data are yet available. Our structured argumentation approach clearly indicates where evidence to support a claim is not yet available: identifying potential areas requiring further investigation. For example, claim 1.1.1.4.2 in figure 3 has a blank diamond appended, showing that it is an undeveloped claim, because there was not, at the time of simulation development, any quantitative biological data on aggregations of haematopoietic cells that we could objectively compare to the simulated aggregations that form *in silico*. A researcher assessing our model can clearly see that we are *not* claiming to produce aggregations of haematopoietic cells that are quantitatively similar to that observed *in vivo*.

### 3.4. Engineering: justifying model abstractions

Concerns over the modelling of a biological system that is incomplete is one criticism often levelled at simulation. In scenarios where this is the case, or the biological knowledge is sufficient yet modelling the full detail is not computationally feasible, abstractions need to be made. The impact these abstractions have on simulation response needs to be analysed and understood, in order that researchers can translate simulation results into meaningful biological hypotheses.

**Figure 4.**
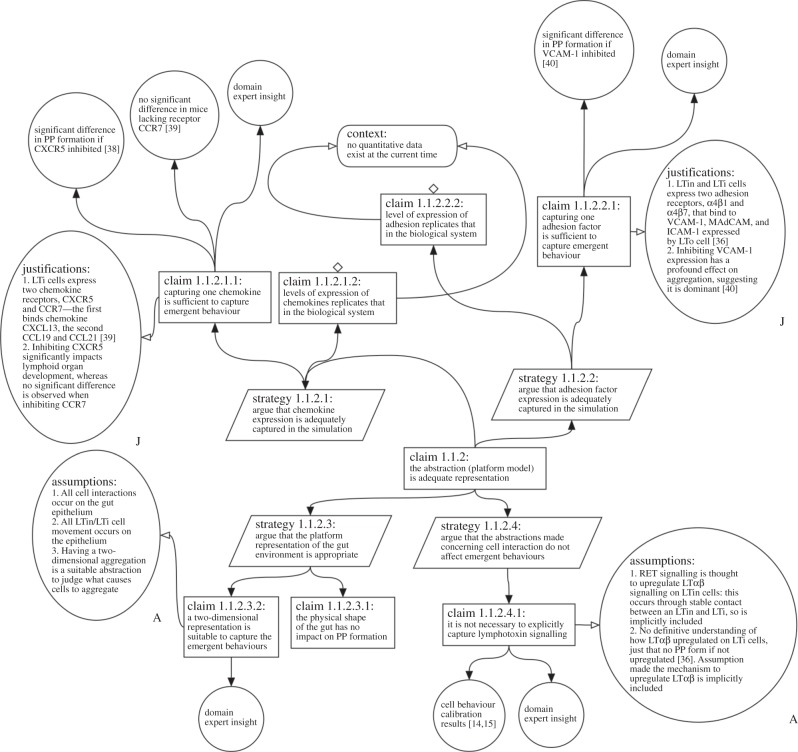
Expansion of claim 1.1.2 in figure 1. This claim examines the abstractions that have been made and whether these are fit for purpose. In this case, the implementation of chemokines, adhesion factors, the intestine environment and cell signalling is explored.

Figure 4 shows the argument supporting claim 1.1.2 of figure 2, that the abstractions made in our model of lymphoid tissue development are appropriate. The abstractions in this specific case had to be made due to an incomplete understanding of chemokine expression and adhesion factor expression. While the generally accepted model of lymphoid tissue development suggests that haematopoietic cell aggregation is driven by the expression of three chemokines binding to two receptors on haematopoietic cells, other published research suggests that only one chemokine causes a significant difference in the formation of cell aggregation [38,39]. As such, we have made the abstraction that a pathway consisting of one chemokine and one receptor is sufficient to capture this mechanism. As detailed in the top left of figure 4, studies in the literature suggest that this may be a suitable abstraction to make, as the inhibition of receptor CXCR5 causes a significant difference in the formation of cell aggregation [38], yet there is no significant difference where the CCR7 receptor is inhibited [39]. Similar abstractions have been drawn for adhesion factor expression, with experimental results suggesting that as one adhesion pathway has a more significant impact on cell aggregation [40], others can be abstracted from the simulation. A stringent analysis of each abstraction has provided us with a method to document these abstractions for full scientific scrutiny, allowed us to justify why these have been made with supporting evidence and to direct researchers assessing our simulation to that available evidence.

### 3.5. Reproducing emergent behaviour: cell aggregation

**Figure 5.**
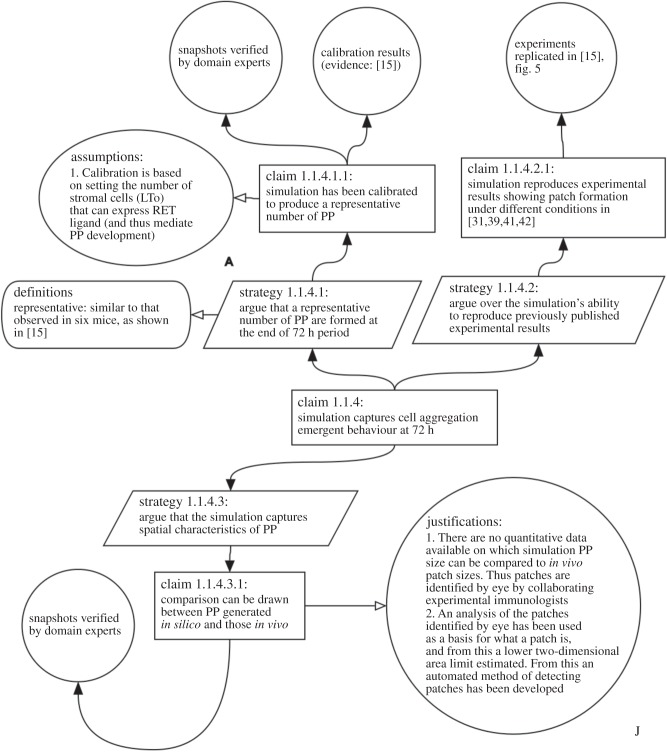
Expansion of claim 1.1.4 in figure 1. This claim states that the simulator appropriately replicates the observed emergent behaviour: cell aggregation indicative of Peyer's Patch formation.

Subclaim 1.1.4 is expanded in figure 5. Here, we are examining whether our simulator reproduces the second emergent behaviour observed in *ex vivo* culture: the formation of aggregations of haematopoietic cells by hour 72 of the process. Our argument is structured over three strategies: determining whether a representative number of aggregations is formed in the intestine environment; exploring whether previously published experimental results that examine aggregations under different physiological conditions are replicated and showing that the simulation captures appropriately the spatial characteristics of aggregations. These claims are more difficult to argue, due to the lack of quantitative biological data available. We can show that our simulator reproduces laboratory gene knockout experiments [31,39,41,42], but determining whether we capture aggregation characteristics appropriately relies on the expert opinion of our collaborating experimental biologists. Again, our inability to objectively support the claims does not represent a failure of the model; the argument clearly shows our claims and evidence, which are open for scrutiny. We contend that, where a simulation is developed to answer a specific question, the more open researchers are about the decisions behind the implementation, the more confidence others have in assessing the impact of simulation-derived results.

A paper description of our argumentation process makes the approach appear static: claims have been made and justified, areas of further development have been identified and supporting documentation produced. However, we use the argumentation process as a dynamic and inherent part of simulation design and results analysis. Argument development and refinement continues as the simulator is analysed and developed further.

## 4. Discussion

Explorations of biological systems have used models, whether biological or computational, to achieve various objectives: to produce hypotheses to inform future laboratory experimentation or clinical trials, to understand complex datasets and to attempt to understand theories that cannot be examined using other methods. Yet, the overriding objective is to interpret model results in the context of the biological domain. To achieve this, the researcher must understand the mapping between the biological system being studied and the model of that system.

Specific to biological simulations, the researcher creating the simulation must understand the impact that decisions made in implementation have on simulation behaviour, and those interpreting the results must understand how these decisions influence what that result means in the context of the real-world system. Although a large amount of work has been undertaken in the development of tools that aid the implementation and analysis of simulations of biological systems, a method for demonstrating that a simulation implementation is an adequate representation of the biology has yet to be adopted. This is challenging given that simulation development should be an inherently cross-discipline activity. Here, we have shown that the application of a structured argumentation approach has the potential to address this, using our previously described lymphoid tissue simulator as an exemplar. By taking safety-critical system engineering as an inspiration, simulation developers can use argumentation to provide a detailed case that their tool is fit for the clearly stated purpose for which it has been designed, supported by available evidence. Where these tools are applied as part of a key decision-making process or for hypothesis generation, such as drug development, a clear rationale for the fitness for purpose of the model is essential.

Conducting a structured analysis of the fitness-for-purpose requirements using GSN produces a diagrammatic summary that can be created and interpreted by researchers across the disciplines, opening the implementation to scrutiny by experimental biologists as well as simulation developers. We have previously advocated a principled approach to simulation development where a number of models are developed: a model consisting exclusively of the biological information to be captured, a translation of this model into a specification that can be encoded as a simulation, an executable of that specification and a model detailing how simulation-derived results will be interpreted in terms of the biological domain [15,43]. By creating argumentation structures at each stage of simulation development, each step in the simulation development is made transparent, the reasoning behind the inclusion or exclusion of a biological feature or assumption is provided and evidence given as to why this conclusion has been drawn. By including descriptions of experiments that have been performed using the simulation, other researchers are equipped with all the detail required to repeat these experiments, to judge their contribution to their own research. The choice of biological data upon which the simulator is run, and how the result has been interpreted, is made explicit. Specific areas where the current biological understanding is lacking are identified (as undeveloped claims), and the abstractions introduced are explained and justified. An effect of the uncovering of incomplete knowledge is viewed as necessarily making a model unfit for purpose: instead, the process of argumentation highlights where biological experimentation might be focused to improve understanding [44]. These aspects have an impact on the result generated by the simulator, and thus will impact how this result is translated in the context of the real-world domain.

The field of safety-critical systems, from which this approach takes its inspiration, has an argumentation-based culture: one which if adopted by those developing simulations of biological systems, could increase the confidence in the application of computational predictive tools [44]. The adoption of such a culture, and such rigour in model design, could also not be restricted to those developing simulations, yet contribute to much wider discussions concerning the relationship between any model and the biological system it represents. The criticisms often directed towards simulation-based models are beginning to be levied towards cell culture and animal models, with studies questioning how representative these traditional biological models are of the system they have been designed to represent: the human [45,46]. The scope of this paper only considers the impact that a structured argumentation process could have on simulation-derived research, yet the scope for impact outside this area should not be underestimated.

To encourage researchers to adopt an argumentation approach, we have released Artoo. We have demonstrated the use of Artoo in the production of the argumentation structures which support the case that our lymphoid tissue development is fit for the purpose for which it was designed: to provide biologically relevant hypotheses as to why cell behaviour becomes statistically different in the vicinity of initial haematopoietic cell aggregations [14]. Through the use of argumentation, we clearly summarize the biological data upon which our simulation is based, the manner in which we have derived assumptions from this and instances where we have made abstractions in the absence of biological data. As predictions are being made that aim to be grounded in the biological domain, this information is vital to researchers intending to understand the impact of our results.
